# Predictive Performance of the Aggregate Index of Systemic Inflammation for Contrast-Induced Nephropathy After PCI in Elderly ACS Patients

**DOI:** 10.3390/medicina62020361

**Published:** 2026-02-11

**Authors:** Çağatay Önal, Cennet Yıldız, Yasin Yüksel, Burak Ayça

**Affiliations:** 1 Department of Cardiology, Özel Gazi Hospital, İzmir 35230, Türkiye; drcagatayonal@hotmail.com; 2 Department of Cardiology, Bakırköy Dr. Sadi Konuk Education and Research Hospital, İstanbul 34147, Türkiye; 3 Department of Cardiology, Private Beylikdüzü Kolan Hospital, İstanbul 34528, Türkiye; dryasinyuksel@gmail.com; 4 Department of Cardiology, Private Medical Point Hospital, İzmir 35575, Türkiye; drburakayca@yahoo.com.tr

**Keywords:** acute coronary syndrome, percutaneous coronary intervention, contrast-induced nephropathy, systemic inflammation, elderly patients

## Abstract

*Background and Objectives*: This study aimed to evaluate the predictive value of the Aggregate Index of Systemic Inflammation (AISI) for contrast-induced nephropathy (CIN) development in elderly patients with acute coronary syndrome (ACS) undergoing PCI. *Materials and Methods*: The study included consecutive patients aged ≥65 years who underwent PCI for ACS between January 2022 and January 2024. The primary endpoint was the occurrence of CIN, defined as an increase in serum creatinine ≥0.5 mg/dL or ≥25% from baseline within 48–72 h after PCI. The AISI was calculated for each patient. *Results*: A total of 437 patients (mean age 73.7 ± 7.2 years, 64.5% male) were included. The overall incidence of CIN was 25.6%. Patients who developed CIN were older, more frequently female, and had lower left ventricular ejection fraction and albumin but higher SYNTAX I scores and baseline creatinine (all *p* < 0.001). AISI demonstrated a significant predictive accuracy for CIN (AUC: 0.709, 95% CI: 0.647–0.771, *p* < 0.001), which was statistically comparable to the Mehran score (AUC: 0.744, *p* = 0.095). In multivariable analysis, AISI emerged as a strong independent predictor of CIN (OR: 1.345, 95% CI: 1.123–1.437, *p* < 0.001), alongside SYNTAX I scores, baseline creatinine, and serum albumin. Notably, AISI retained its independent predictive power even when adjusted for the Mehran score (OR: 1.276, *p* < 0.001). *Conclusions*: AISI independently predicts CIN in elderly patients with ACS undergoing PCI. Its superior discriminative ability compared with single hematologic markers highlights the importance of systemic inflammatory burden in CIN pathogenesis. Incorporating AISI into pre-procedural assessment may improve risk stratification and preventive management in this high-risk population.

## 1. Introduction

Percutaneous coronary intervention (PCI) plays a pivotal role in the management of acute coronary syndrome (ACS) not only by rapidly restoring coronary perfusion and minimizing myocardial damage but also by improving both short- and long-term outcomes [1,2]. Advances in stent design, antiplatelet therapy, and intravascular imaging have further enhanced PCI safety and efficacy, making it the cornerstone of contemporary ACS treatment strategies [3,4]. Contrast-induced nephropathy (CIN), defined as an absolute increase in serum creatinine of ≥0.5 mg/dL or a relative increase of ≥25% from the baseline value within 48–72 h after the procedure, remains one of the most frequent and clinically significant complications following PCI, particularly among patients with ACS [5]. The combination of hemodynamic instability, a high thrombotic burden, and the need for multiple contrast injections predisposes ACS patients to renal injury, resulting in longer hospitalization and higher short- and long-term mortality rates [6,7,8,9]. Even mild elevations in serum creatinine after PCI have been linked to adverse cardiovascular outcomes such as all-cause mortality, ACS, heart failure hospitalization, or stroke [6]. Several factors contribute to CIN pathogenesis, including renal vasoconstriction, oxidative stress, and direct tubular toxicity of iodinated contrast media [9]. Increasing evidence have indicated that inflammation plays a central role in the pathogenesis of CIN. Besides renal vasoconstriction and direct tubular cytotoxicity, contrast exposure triggers endothelial dysfunction, oxidative stress, and activation of inflammatory pathways that exacerbate renal injury [10,11]. Contrast agents stimulate the release of pro-inflammatory cytokines, leukocyte infiltration and microvascular dysfunction [12]. Several studies have shown that elevated inflammatory indices—such as the neutrophil-to-lymphocyte ratio and the systemic immune–inflammation index, are independently associated with an increased risk of CIN after PCI [13,14].

Aging is associated with a progressive decline in renal functional reserve, an increased prevalence of chronic comorbidities, and structural renal changes, which heighten susceptibility to contrast-related renal injury and are consistent with the higher frequency of early adverse clinical outcomes, including heart failure, cardiogenic shock, premature mortality, and cerebrovascular events, observed in elderly patients with ST-elevation acute myocardial infarction [15,16]. Previous studies have consistently demonstrated that elderly individuals have a significantly higher incidence of CIN and worse clinical outcomes, including longer hospitalization, higher rates of heart failure, and increased short- and long-term mortality [17,18]. Most studies investigating CIN after PCI have included patients across all age groups, in which age was treated merely as a covariate rather than the primary variable of interest. Separate subgroup analyses focusing exclusively on elderly patients are relatively scarce. Moreover, large multicenter trials have excluded frail elderly individuals or those with multiple comorbidities who represent a substantial portion of real-world clinical practice.

Recent evidence continues to highlight the prognostic importance of composite inflammatory biomarkers, which offer superior predictive accuracy compared to single-cell markers by integrating various pathways of the immune response. Specifically, these indices provide a more comprehensive reflection of the systemic inflammatory burden, making them powerful tools for risk stratification in acute cardiovascular settings [19,20]. The Aggregate Index of Systemic Inflammation (AISI) has emerged as a novel and integrative marker that potentially captures the complex interplay between innate and adaptive immunity more effectively than single-cell parameters, particularly in high-risk populations such as the elderly. In the present study, we specifically aimed to investigate the predictive role of the AISI in the development of CIN among elderly patients with ACS undergoing PCI.

## 2. Materials and Methods

This single-center, retrospective cohort study was conducted to investigate the development of CIN in elderly patients undergoing PCI for ACS. Consecutive patients who underwent PCI at our tertiary care center between 1 January 2022, and 1 January 2024, were screened for eligibility, and only those aged 65 years or older were included in the final analysis. All procedures were performed in accordance with the principles of the Declaration of Helsinki, and the study protocol was approved by the local ethics committee.

Patients were eligible for inclusion if they met the following criteria: a confirmed diagnosis of ACS, including ST-elevation myocardial infarction (STEMI), non–ST-elevation myocardial infarction (NSTEMI), or unstable angina pectoris (UAP); undergoing index PCI during the same hospitalization; availability of baseline serum creatinine measurement before PCI and at least one follow-up creatinine value obtained within 48–72 h after the procedure; and availability of complete blood count parameters within 24 h prior to PCI. The diagnosis of ACS was established in accordance with current international guidelines based on clinical presentation, electrocardiographic findings, and cardiac biomarker levels [1,2]. Patients were excluded if they had dialysis-dependent chronic kidney disease, acute infection, active malignancy, hematologic disorders or known chronic systemic inflammatory diseases (e.g., rheumatoid arthritis, systemic lupus erythematosus); were receiving systemic corticosteroids or other immunosuppressive therapies; had hemodynamically significant bleeding or required blood transfusion during hospitalization; developed acute kidney injury due to non–contrast-related causes such as shock or sepsis; or had incomplete clinical or laboratory data. Clinical and demographical data of the patients were obtained from hospital data system.

The primary endpoint of the study was the development of CIN, which was defined as an increase in serum creatinine of ≥0.5 mg/dL or ≥25% from the baseline value within 48–72 h after the PCI procedure, in accordance with the criteria of the European Society of Urogenital Radiology [5]. AISI was calculated for each patient using absolute peripheral blood counts obtained at admission according to the following formula:

AISI = (neutrophils × monocytes × platelets)/lymphocytes

Additionally, the Mehran scoring system, comprising eight distinct clinical and procedural variables, was calculated for each patient. These variables included the presence of hypotension, use of an intra-aortic balloon pump, congestive heart failure, advanced age (>75 years), anemia, diabetes mellitus, contrast media volume, and baseline kidney function (estimated glomerular filtration rate). The total risk score was computed by summing the pre-specified weights assigned to each factor [21]. In accordance with institutional standards, isotonic saline hydration at a rate of 1–1.5 mL/kg/hour was administered to eligible patients for 3–12 h before and 6–24 h after the procedure.

### Statistical Analysis

Based on an expected CIN prevalence of 20%, the minimum required sample size was calculated as approximately 246 patients, assuming a 95% confidence level and a margin of error of ±5%. Descriptive statistics were expressed as mean ± standard deviation for continuous variables, and as frequency (percentage) for categorical variables. The normality of distribution was assessed using the Shapiro–Wilk test. For group comparisons between patients with and without CIN, the independent samples *t*-test or Mann–Whitney U test was applied for continuous variables, as appropriate, and the chi-square or Fisher’s exact test for categorical variables.

Receiver operating characteristic (ROC) curve analysis was performed to evaluate and compare the diagnostic performance of the AISI and the Mehran score in predicting CIN. The area under the curve (AUC), along with corresponding 95% confidence intervals (CI) and *p*-values, was calculated for each tool. Pairwise AUC comparisons between the AISI and the Mehran score were conducted using the DeLong test to determine whether the observed differences in their predictive performances were statistically significant. Univariate and multivariable logistic regression analyses were performed to identify factors associated with the development of CIN. Variables with a *p*-value < 0.05 in the univariate analysis were subsequently included in the multivariable models. Two separate models were constructed to determine independent predictors of CIN in the multivariable analysis. Model A included age, gender, cardiogenic shock, left ventricular ejection fraction (LVEF), Synergy Between Percutaneous Coronary Intervention with TAXUS and Cardiac Surgery (SYNTAX) Score I, baseline serum creatinine, serum albumin, maximum troponin, hemoglobin, neutrophil count, and lymphocyte count. In Model B, neutrophil and lymphocyte counts were replaced by the AISI as a composite inflammatory marker. The results of both univariate and multivariable logistic regression analyses were expressed as odds ratios (OR) with corresponding 95% confidence intervals (CI). Multicollinearity was assessed before model construction, and a two-tailed *p*-value < 0.05 was considered statistically significant. All statistical analyses were conducted using IBM SPSS Statistics version 25 (IBM Corp., Armonk, NY, USA).

## 3. Results

The mean age of the entire cohort was 73.67 ± 7.20 years, and 64.5% (n = 282) were male. The overall incidence of CIN was 25.62% (n = 112). Patients who developed CIN were significantly older than those without CIN (*p* < 0.001) and more frequently female (*p* = 0.034). CIN-positive patients had a lower left ventricular ejection fraction (LVEF) (*p* < 0.001) and a higher incidence of cardiogenic shock (*p* < 0.001). The SYNTAX I score was significantly higher in the CIN (+) group, indicating greater angiographic complexity (*p* < 0.001). Baseline and post-procedural serum creatinine levels were both elevated in CIN-positive patients, while baseline glomerular filtration rate (GFR) was significantly lower (*p* < 0.001 for all). Inflammatory and nutritional indices also differed notably between groups. CIN (+) patients had lower serum albumin, lymphocyte and hemoglobin (*p* < 0.001 for all) values, but higher neutrophil (*p* < 0.001) and monocyte (*p* = 0.001) counts. AISI was markedly elevated in the CIN (+) group (*p* < 0.001). Patients who developed CIN exhibited a significantly greater mean Mehran score than those who did not (*p* < 0.001). The volume of contrast media administered did not differ significantly between the CIN (+) and CIN (−) groups (208.25 ± 85.27 mL vs. 204.08 ± 87.24 mL, respectively; *p* = 0.436). Additionally, mechanical circulatory support devices, such as an Impella or intra-aortic balloon pump, were not utilized in any of the study patients. (Table 1).

**Figure 1:**
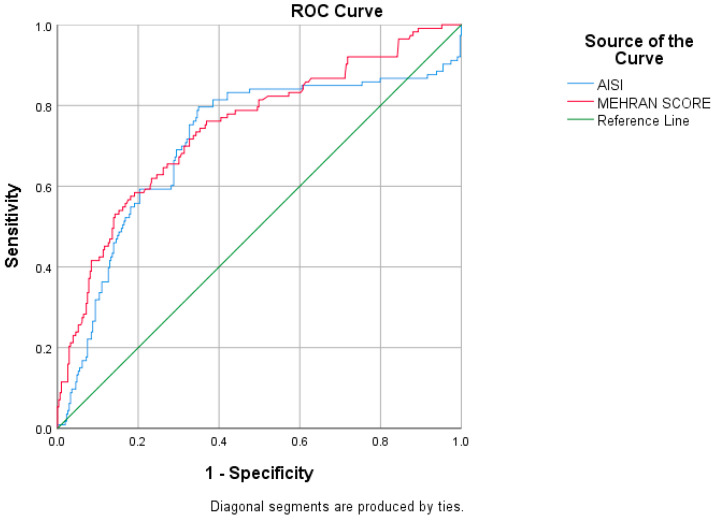
Comparison of ROC curves for the Mehran score and AISI in predicting contrast-induced nephropathy (CIN).

The Mehran score demonstrated an AUC of 0.744 (95% CI: 0.689–0.799, *p* < 0.001), while the AISI yielded an AUC of 0.709 (95% CI: 0.647–0.771, *p* < 0.001) (Figure 1). Although the Mehran score showed a numerically higher predictive value, the pairwise comparison of the two ROC curves using the DeLong test revealed no statistically significant difference between their diagnostic performances. The estimated difference in AUC was 0.035 (95% CI: −0.006 to 0.076; z = 1.667; *p* = 0.095).

In the univariable logistic regression analysis (Table 2), several clinical and laboratory parameters were found to be significantly associated with the development of CIN. Older age and male gender emerged as significant demographic predictors. Among the clinical variables, the presence of cardiogenic shock, the Mehran score, and higher SYNTAX Score I were identified as strong predictors of CIN, whereas LVEF demonstrated an inverse association. Baseline serum creatinine and maximum troponin levels were also significantly related to the occurrence of CIN. In contrast, serum albumin and hemoglobin levels were negatively associated with CIN, suggesting their potential protective roles. Among inflammatory markers, both neutrophil count and the AISI were significantly associated with an increased risk of CIN, whereas lymphocyte count exhibited an inverse relationship.

In the multivariable logistic regression analysis (Table 3), three distinct models were constructed to identify independent predictors of CIN.

Model A analyzed individual clinical and laboratory parameters, including age, gender, cardiogenic shock, LVEF, SYNTAX Score I, baseline serum creatinine, serum albumin, maximum troponin, hemoglobin, and individual leukocyte counts. In this model, SYNTAX Score I (OR: 1.100, 95% CI: 1.050–1.153, *p* < 0.001), baseline serum creatinine (OR: 4.204, 95% CI: 1.915–9.226, *p* < 0.001), and neutrophil count (OR: 1.087, 95% CI: 1.025–1.134, *p* = 0.004) were independently associated with an increased risk of CIN. Conversely, lymphocyte count (OR: 0.258, 95% CI: 0.150–0.444, *p* < 0.001) and serum albumin (OR: 0.121, 95% CI: 0.047–0.307, *p* < 0.001) demonstrated protective associations.

In Model B, individual neutrophil and lymphocyte counts were replaced by the AISI to capture the combined inflammatory burden. Similar to Model A, SYNTAX Score I (OR: 1.104, 95% CI: 1.053–1.157, *p* < 0.001), baseline serum creatinine (OR: 4.256, 95% CI: 1.980–9.150, *p* < 0.001), and serum albumin (OR: 0.116, 95% CI: 0.046–0.295, *p* < 0.001) remained independent predictors. Importantly, AISI emerged as a strong and significant predictor of CIN (OR: 1.345, 95% CI: 1.123–1.437, *p* < 0.001), suggesting that this composite inflammatory index provides superior explanatory power over individual leukocyte components.

Finally, Model C was designed to evaluate the independent predictive value of the Mehran score alongside AISI and other non-overlapping variables. To avoid multicollinearity, individual components of the Mehran score (age, creatinine, hemoglobin, LVEF, and shock) were excluded from this model. The analysis revealed that the Mehran score (OR: 1.826, 95% CI: 1.243–3.321, *p* < 0.001) and AISI (OR: 1.276, 95% CI: 1.134–1.545, *p* < 0.001) were both robust independent predictors of CIN, together with SYNTAX Score I (OR: 1.112, *p* < 0.001) and serum albumin (OR: 0.166, *p* < 0.001). These findings indicate that AISI provides incremental prognostic information that is independent of traditional clinical risk scores.

## 4. Discussion

In the present study, we demonstrated that the AISI is a robust and independent predictor of CIN. Among the evaluated inflammatory markers, AISI exhibited the highest discriminative ability, surpassing individual leukocyte components. Both univariate and multivariable logistic regression analyses confirmed a consistent and independent association between elevated AISI levels and the development of CIN. Notably, in our multivariable models, AISI demonstrated superior predictive power compared to the neutrophil count alone. Furthermore, even when adjusted for the Mehran score—the gold-standard clinical risk stratification tool—AISI remained a significant independent predictor (OR: 1.276, *p* < 0.001). This suggests that the systemic inflammatory burden, as captured by the composite AISI, reflects a distinct pathophysiological pathway not fully accounted for by traditional risk factors. These findings indicate that AISI offers significant clinical relevance and incremental prognostic value, providing a practical and powerful tool for predicting CIN among elderly patients undergoing PCI.

Evidence showed that systemic inflammation plays a crucial role in the pathogenesis of CIN. Inflammatory activation triggers endothelial dysfunction, oxidative injury, and microcirculatory impairment, which collectively increase renal susceptibility to contrast-related ischemic and toxic damage. Activated leukocytes and platelets release cytokines and reactive oxygen species, further aggravating tubular and vascular injury [22]. Earlier research has shown that indices such as the neutrophil-to-lymphocyte ratio, platelet-to-lymphocyte ratio, and systemic immune inflammation index are significantly related to CIN occurrence [23,24,25]. In a recent study by Unkun et al., the prognostic value of AISI for predicting contrast-induced acute kidney injury was evaluated in 166 patients who underwent coronary angiography. The incidence of contrast-induced acute kidney injury was 15.1%, and was an independent predictor of renal injury [26]. Although these investigations have provided valuable insights into the association between systemic inflammation and CIN, they were conducted in mixed-age populations, in which advanced age was treated merely as an adjustment variable rather than a primary determinant. However, older adults differ markedly from younger individuals in terms of the inflammatory response, renal autoregulation, and susceptibility to contrast-induced renal injury. Age-related endothelial dysfunction, microvascular fragility, and the chronic low-grade inflammatory state may amplify the deleterious effects of contrast agents [27,28]. In our cohort, the incidence of CIN was found to be 25.6%. This rate is noticeably higher than those reported in previous studies conducted in general adult populations, where CIN incidence typically ranges between 10% and 15%, suggesting that advanced age substantially amplifies vulnerability [23,25,26]. Moreover, in our study AISI remained a strong and independent predictor of CIN even in an elderly population, indicating that the prognostic value of AISI is not limited to general adult cohorts but also extends to older patients who represent a high-risk subset in real-world clinical practice. AISI demonstrated superior predictive performance for CIN compared with single inflammatory components such as neutrophil, platelet, monocyte, or albumin levels. Unlike isolated parameters that reflect only one dimension of the inflammatory response, AISI integrates cellular elements of both innate and adaptive immunity, thereby providing a more comprehensive assessment of systemic inflammatory burden. This integrative nature likely explains its stronger association with renal outcomes, as CIN is a multifactorial process influenced by oxidative stress, endothelial dysfunction, and microvascular inflammation.

One of the most significant findings of our study was the independent association of AISI with CIN, even when the model was adjusted for the Mehran score. As the most widely recognized clinical risk stratification tool, the Mehran score incorporates established factors such as age, baseline renal function, and hemodynamic status. However, our multivariable analysis (Model C) demonstrated that AISI remained a robust predictor alongside the Mehran score. By integrating AISI into the clinical evaluation, we can capture a distinct pathophysiological dimension—systemic inflammatory burden—that provides incremental prognostic value. Therefore, in the high-risk elderly population, the combined use of the Mehran score and AISI may offer a more nuanced and accurate risk assessment for the development of CIN.

Regarding clinical implementation, AISI serves as a comprehensive tool for pre-procedural risk stratification. While several formulas currently exist to calculate maximum contrast dose based on renal function, a specific ‘AISI-based’ contrast limit formula has not yet been defined in the literature [29]. However, our findings suggest that an AISI value exceeding the 677.12 cut-off identifies a high-risk phenotype that requires more vigilant preventive measures. In routine practice, patients with high AISI levels may benefit from more conservative contrast dosing, the use of ultra-low contrast volumes, and intensified peri-procedural hydration protocols. Future investigations should focus on whether integrating AISI into existing risk scores improves their predictive accuracy and whether AISI-guided hydration protocols can directly reduce the incidence of CIN in the elderly population.

Several other findings of our study also merit discussion. In our multivariable analysis, higher SYNTAX Score I, elevated baseline serum creatinine, and lower serum albumin emerged as independent predictors of CIN. Higher SYNTAX scores reflects greater coronary lesion complexity and higher systemic atherosclerotic burden, necessitating longer procedures and increased contrast exposure, all of which contribute to renal hypoperfusion and contrast-induced injury [30]. Baseline elevated creatinine indicates diminished renal reserve, making kidneys less capable of tolerating the nephrotoxic and ischemic effects of iodinated contrast—a well-recognized risk factor in CIN pathophysiology [31,32,33]. Hypoalbuminemia may reflect subclinical inflammation, malnutrition or impaired antioxidant capacity, thereby facilitating damage in the renal parenchyma after contrast exposure; previous studies have similarly identified low serum albumin as a risk factor for CIN and adverse renal outcomes [34,35]. Our findings underscored the importance of comprehensive pre-procedural risk assessment in older patients.

## 5. Conclusions

AISI independently predicted the occurrence of CIN in elderly patients with ACS undergoing PCI. It provided a more comprehensive representation of the systemic immune response, demonstrating stronger discriminative ability for CIN risk. The high incidence of CIN observed in our elderly cohort underscores the increased age-related susceptibility to contrast-induced renal injury. Greater coronary lesion complexity, impaired renal function, and poorer nutritional status collectively influenced renal outcomes. Notably, the prognostic value of AISI remains significant even when adjusted for the Mehran risk score, suggesting that it captures a distinct inflammatory dimension of renal risk. Our findings suggest that incorporating AISI into routine pre-procedural risk assessment, alongside established clinical and anatomical scores, could lead to more precise risk stratification and tailored preventive strategies for elderly patients.

## 6. Limitations

First, the study had retrospective, single-center design. Second, inflammatory indices were derived from single pre-procedural measurements, and temporal variations in inflammatory status could not be assessed. Third, our analysis was specifically designed to evaluate in-hospital renal outcomes (CIN), and major adverse cardiovascular events were not evaluated. Consequently, whether the AISI predicts other clinical cardiovascular outcomes in this specific cohort remains to be determined in future prospective studies with longer follow-up periods. Finally, although we adjusted for several confounders, including the Mehran score, the potential influence of unmeasured variables cannot be entirely excluded.

## Figures and Tables

**Table 1:** Baseline clinical characteristics of the total study population and groups with and without contrast-induced nephropathy (CIN).

	Study Population (n = 437)	CIN (−) (n = 325)	CIN (+) (n = 112)	*p* Value
Variable				
Age (years)	73.67 ± 7.20	72.63 ± 6.30	76.67 ± 8.67	<0.001
Gender, n (%)				0.034
•Male	282 (64.5)	219 (67.4)	63 (56.3)	
•Female	155 (35.5)	106 (32.6)	49 (43.8)	
BMI (kg/m^2^)	27.57 ± 5.34	27.51 ± 4.71	27.73 ± 6.53	0.411
Diabetes mellitus, n (%)	156 (35.7)	110 (33.9)	46 (41.1)	0.169
Hypertension, n (%)	391 (69.5)	286 (88)	105 (93.8)	0.072
Hyperlipidemia, n (%)	363 (83.1)	267 (82.2)	96 (85.7)	0.386
ACEI/ARB use, n (%)	330 (75.5)	250 (76.9)	80 (71.4)	0.244
Beta-blocker use, n (%)	337 (85.1)	249 (85.3)	88 (84.6)	0.871
Calcium channel blocker use, n (%)	120 (30.3)	64 (21.9)	58 (53.8)	
Diuretic use, n (%)	158 (36.2)	112 (34.5)	46 (41.1)	0.209
Statin use, n (%)	192 (48.5)	134 (45.9)	58 (55.8)	0.083
ASA use, n (%)	337 (85.1)	251 (86)	86 (82.7)	0.422
ACS type, n (%)				0.706
•NSTEMI	235 (53.8)	175 (53.8)	60 (53.6)	
•STEMI	163 (37.3)	119 (36.6)	44 (39.3)	
•UAP	39 8.9)	31 (9.5)	8 (7.1)	
Re-infarction, n (%)	35 (8.4)	23 (7.3)	12 (11.5)	0.190
Cardiogenic shock, n (%)	14 (3.2)	3 (0.9)	11 (9.8)	<0.001
LVEF (%)	50.95 ± 9.79	52.86 ± 9.11	45.43 ± 9.63	<0.001
Smoking status, n (%)				0.061
•Current smoker	115 (26.3)	94 (28.9)	21 (18.8)	
•Ex-smoker	79 (18.1)	60 (18.5)	19 (17)	
Scope time (min)	17.38 ± 6.53	17.17 ± 6.58	18.01 ± 6.38	0.070
Contrast volume (mL)	205.14 ± 86.66	204.08 ± 87.24	208.25 ± 85.27	0.436
SYNTAX Score I	13.42 ± 8.75	10.90 ± 6.42	20.71 ± 10.41	<0.001
Baseline serum creatinine (mg/dL)	0.99 ± 0.41	0.91 ± 0.31	1.20 ± 0.54	<0.001
Post-procedural serum creatinine (mg/dL)	1.29 ± 0.82	0.96 ± 0.22	2.23 ± 1.15	<0.001
Baseline estimated GFR (mL/min/1.73 m^2^)	73.66 ± 21.39	80.47 ± 16.12	53.96 ± 22.53	<0.001
LDL-C (mg/dL)	119.11 ± 41.32	121.29 ± 42.31	113.01 ± 37.95	0.101
Triglycerides (mg/dL)	143.12 ± 71.49	141.48 ± 70.21	147.74 ± 75.11	0.610
HDL-C (mg/dL)	43.40 ± 19.85	44.33 ± 12.08	40.81 ± 33.24	0.122
Serum albumin (g/dL)	3.89 ± 0.50	4.05 ± 0.41	3.44 ± 0.44	<0.001
Maximum troponin (ng/mL)	8615.26 ± 13,133.07	7639 ± 13,351.47	11,446.71 ± 12,094.92	<0.001
Hemoglobin (g/dL)	12.60 ± 2.01	12.97 ± 1.91	11.50 ± 1.87	<0.001
Neutrophil count (×10^3^/µL)	6.66 ± 3.42	6.05 ± 3.41	8.45 ± 2.77	<0.001
Platelet count (×10^3^/µL)	256.10 ± 90.23	252.82 ± 92.33	265.60 ± 83.51	0.085
Lymphocyte count (×10^3^/µL)	1.96 ± 0.90	2.17 ± 0.86	1.37 ± 0.72	<0.001
Monocyte count (×10^3^/µL)	0.67 ± 0.26	0.66 ± 0.25	0.72 ± 0.28	0.001
AISI	910.82 ± 1179.30	602.44 ± 702.01	1805.89 ± 1714.00	<0.001
Mehran score	6.91 ± 4.28	5.74 ± 3.56	10.35 ± 4.37	<0.001

ACEI = angiotensin-converting enzyme inhibitor; ACS = acute coronary syndrome; ARB = angiotensin receptor blocker; ASA = acetylsalicylic acid; BMI = body mass index; CIN = contrast-induced nephropathy; GFR = glomerular filtration rate; HDL-C = high-density lipoprotein cholesterol; LDL-C = low-density lipoprotein cholesterol; LVEF = left ventricular ejection fraction; NSTEMI = non–ST-elevation myocardial infarction; STEMI = ST-elevation myocardial infarction; UAP = unstable angina pectoris.

**Table 2:** Univariable logistic regression analysis for prediction of CIN.

	*p*	OR	95% CI
Age	<0.001	1.078	1.046–1.110
Gender	0.034	0.622	0.401–0.966
Cardiogenic shock	<0.001	11.690	3.199–42.723
LVEF	<0.001	0.926	0.905–0.948
SYNTAX Score I	<0.001	1.154	1.117–1.192
Baseline serum creatinine	<0.001	7.390	3.401–16.060
Serum albumin	<0.001	0.048	0.024–0.094
Maximum troponin	0.012	1.001	1.001–1.002
Hemoglobin	<0.001	0.675	0.598–0.761
Neutrophil count	<0.001	1.194	1.180–1.407
Lymphocyte count	<0.001	0.225	0.153–0.331
Monocyte count	0.052	2.221	0.993–4.967
AISI	<0.001	1.234	1.123–1.345
Mehran score	<0.001	2.143	1.543–4.921

AISI = Aggregate Index of Systemic Inflammation; CI = Confidence Interval; LVEF = Left Ventricular Ejection Fraction; OR = Odds Ratio; SYNTAX Score I = Synergy Between PCI With Taxus and Cardiac Surgery Score I.

**Table 3:** Multivariable logistic regression analysis for prediction of CIN.

**MODEL A**			
	***p***	**OR**	**95% CI**
Age	0.405	1.021	0.973–1.071
Gender	0.141	0.565	0.264–1.208
Cardiogenic shock	0.815	1.256	0.186–8.497
LVEF	0.382	0.982	0.942–1.023
SYNTAX Score I	<0.001	1.100	1.050–1.153
Baseline serum creatinine	<0.001	4.204	1.915–9.226
Serum albumin	<0.001	0.121	0.047–0.307
Maximum troponin	0.686	1.000	1.000–1.000
Hemoglobin	0.618	0.949	0.774–1.165
Neutrophil count	0.004	1.087	1.025–1.134
Lymphocyte count	<0.001	0.258	0.150–0.444
**MODEL B**			
Age	0.304	1.025	0.977–1.076
Gender	0.360	0.715	0.348–1.467
Cardiogenic shock	0.928	1.092	0.161–7.399
LVEF	0.402	0.983	0.944–1.023
SYNTAX Score I	<0.001	1.104	1.053–1.157
Baseline serum creatinine	<0.001	4.256	1.980–9.150
Serum albumin	<0.001	0.116	0.046–0.295
Maximum troponin	0.526	1.000	1.000–1.000
Hemoglobin	0.186	0.878	0.724–1.065
AISI	<0.001	1.345	1.123–1.437
**MODEL C**			
Gender	0.250	0.668	0.336–1.328
SYNTAX Score I	<0.001	1.112	1.065–1.161
Serum albumin	<0.001	0.166	0.071–0.385
Maximum troponin	0.921	1.001	1.001–1.002
AISI	<0.001	1.276	1.134–1.545
Mehran score	<0.001	1.826	1.243–3.321

CI = Confidence Interval; CIN = Contrast-Induced Nephropathy; LVEF = Left Ventricular Ejection Fraction; OR = Odds Ratio; SYNTAX Score I = Synergy Between PCI With Taxus and Cardiac Surgery Score I; AISI = Aggregate Index of Systemic Inflammation.

## Data Availability

The data supporting the findings of this study are not publicly available due to ethical and privacy restrictions, as they contain sensitive patient information. The data are available from the corresponding author upon reasonable request and with the permission of the Non-Interventional Clinical Research Ethics Committee of İzmir Bakırçay University.

## Abbreviations

The following abbreviations are used in this manuscript:

ACS	Acute Coronary Syndrome
AISI	Aggregate Index of Systemic Inflammation
CI	Confidence Interval
CIN	Contrast-Induced Nephropathy
GFR	Glomerular Filtration Rate
LVEF	Left Ventricular Ejection Fraction
MACE	Major Adverse Cardiac Events
NSTEMI	Non–ST-Elevation Myocardial Infarction
OR	Odds Ratio
PCI	Percutaneous Coronary Intervention
ROC	Receiver Operating Characteristic
STEMI	ST-Elevation Myocardial Infarction
SYNTAX	Synergy Between Percutaneous Coronary Intervention with TAXUS and Cardiac Surgery
UAP	Unstable Angina Pectoris
